# A Rare Case of Dislodged Chemoport Catheter Entrapment in the Pulmonary Artery

**DOI:** 10.1155/crvm/9100288

**Published:** 2025-03-19

**Authors:** Sanjay Shrestha, Naveen Kumar Pandey, Lokesh Shekher Jaiswal, Achyut Gyawali, Brijesh Pandey, Rajan Thapa, Jeet Prasad Ghimire, Bhuwan Thapa, Pawan Chaurasia

**Affiliations:** ^1^Department of Cardiology, BP Koirala Institute of Health Sciences, Dharan, Nepal; ^2^Department of Cardiothoracic and Vascular Surgery, BP Koirala Institute of Health Sciences, Dharan, Nepal

## Abstract

Implantable subcutaneous chemoports are routinely employed for delivering chemotherapy in oncology. Spontaneous catheter dislodgement and embolization of the catheters are rare complications of the procedure. Herein, we report our experience with a patient presenting with spontaneous dislodgement and migration of the catheter to the pulmonary artery. The patient having familial adenomatous polyposis with adenocarcinoma of the right colon underwent total proctocolectomy and had placement of the chemoport through the internal jugular vein for adjuvant FOLFOX chemotherapy. The entrapped catheter was successfully managed by percutaneous retrieval by an interventional cardiologist.

## 1. Introduction

Central venous access devices are extensively used in patients with oncological diseases to deliver chemotherapy. Implantable subcutaneous chemoports are easy to implant under local anesthesia as a daycare treatment and can provide less discomfort, are inexpensive, and enable ambulatory maintenance [1, 2]. Spontaneous fracture of the catheter and migration of a catheter fragment are rare complications, with the incidence of catheter fracture varying from 0.1% to 1.8% [1, 3, 4]. The dislodged catheter may embolize or migrate to the vena cava, right atrium, right ventricle, or the pulmonary artery and its branches [5, 6]. We report a case of spontaneous dislodgement of the port catheter and its migration into the pulmonary artery, managed with percutaneous retrieval.

## 2. Case Report

A 52-year-old male was diagnosed as having familial adenomatous polyposis with adenocarcinoma of the right colon, underwent total proctocolectomy, and was subsequently planned for adjuvant chemotherapy with an FOLFOX regimen in our hospital.

**Figure 1:**
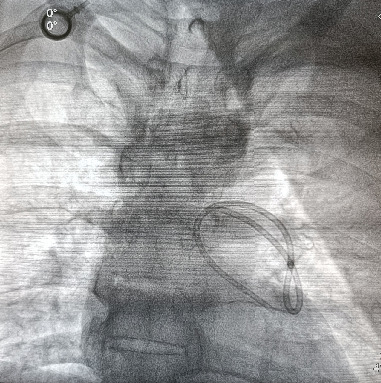
Cinefluoroscopic view of the dislodged and migrated catheter (AP view).

**Figure 2:**
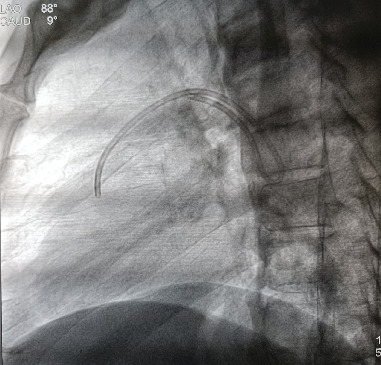
Cinefluoroscopic view of the dislodged and migrated catheter (LAO caudal).

A chemoport catheter (HealthPORT venous PLP All-in-1S-8FR, Plan1Health s.r.l.) was placed in the right internal jugular vein, and the port was placed in the chest wall for chemotherapy. A chest x-ray after the procedure revealed good placement of the chemoport catheter. Then he underwent four cycles of chemotherapy in the next 2 months through this port and was responding well. The patient was planned for the fifth cycle of chemotherapy. On examination, the chemotherapy port was found in situ, but the catheter was not palpable in the subcutaneous tissue of the neck. A chest radiograph and cine fluoroscopic evaluation showed that the chemoport catheter had migrated from its original location to the pulmonary artery (Figures 1 and 2). The patient, however, was asymptomatic with no significant ECG changes. A cardiology consultation was done for possible percutaneous retrieval of the migrated catheter.

As the patient was asymptomatic and stable, an elective procedure was planned. On the next day, the patient was taken to the cath lab, and retrieval was done under fluoroscopic guidance under local anesthesia.

**Figure 3:**
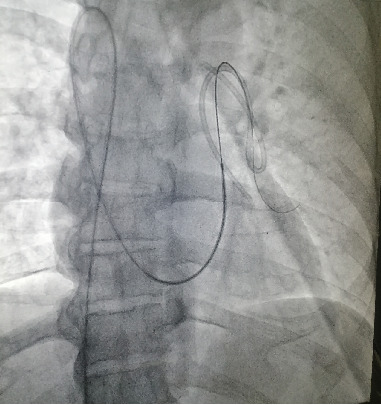
MP catheter in the pulmonary artery.

**Figure 4:**
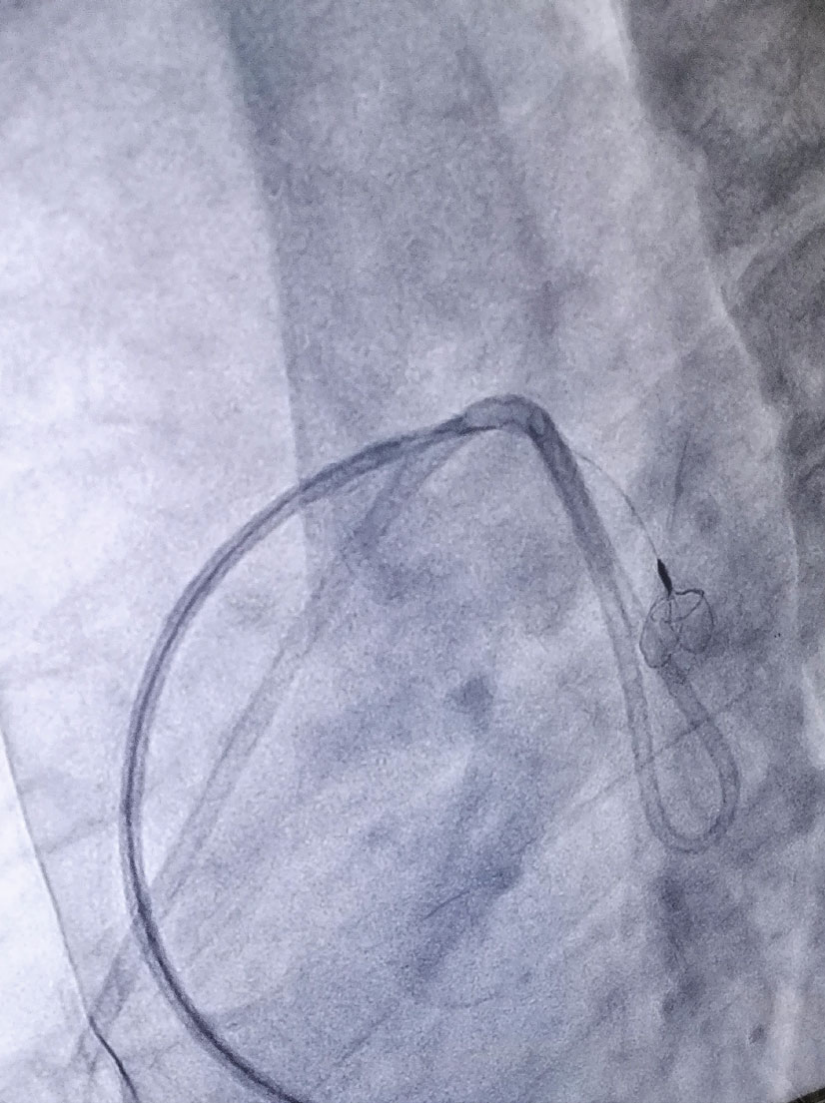
Dislodged catheter being snared.

**Figure 5:**
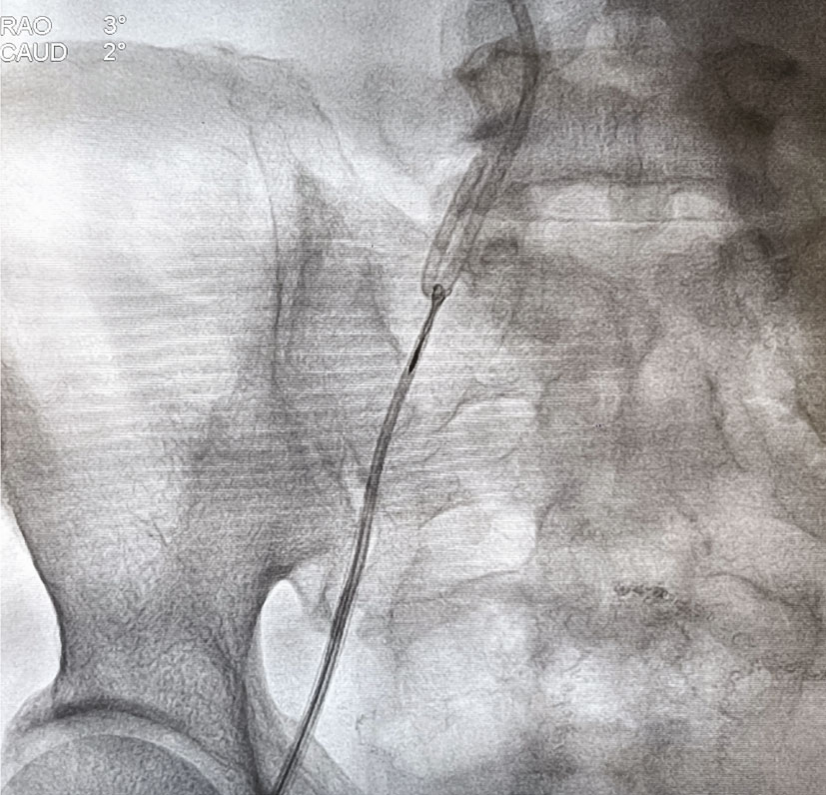
Dislodged catheter being pulled out.

The right femoral vein was punctured, and a seven French sheath was inserted. Percutaneous retrieval of the migrated catheter was performed with a 6 Fr multipurpose catheter and a 3.2 French EN Snare (Figures 3 and 4). The floating end of the migrated catheter in the main pulmonary artery was snared and retrieved via the inferior vena cava towards the right femoral vein under fluoroscopic guidance (Figure 5). The dislodged catheter fragment, along with the retrieval set, was removed through the right femoral vein.

**Figure 6:**
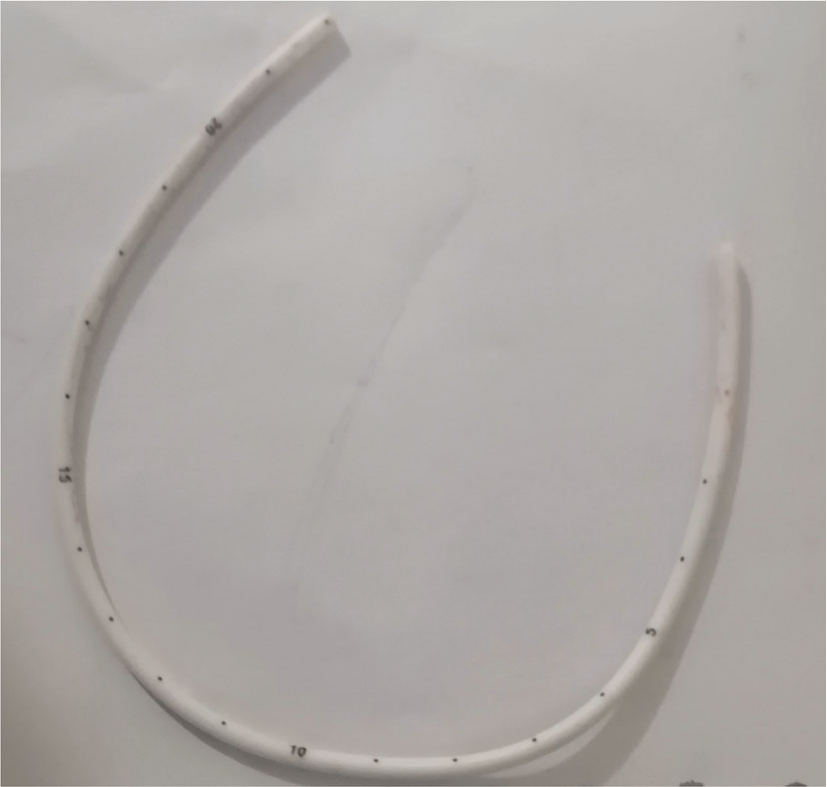
Retrieved chemoport catheter.

The length of the retrieved catheter was 22 cm, and no thrombus was observed at the tip (Figure 6). No major complication occurred during and after the procedure, and the patient was discharged on the same day.

This is a unique case because spontaneous dislodgement of the venous access devices and migration to the pulmonary artery in an asymptomatic patient is very rare, and this is possibly the first case reported from Nepal, with successful percutaneous retrieval of the chemoport catheter from the pulmonary artery.

## 3. Discussion

Subcutaneous infusion ports, commonly employed for chemotherapy, may present with various complications. Delayed complications include spontaneous intravascular fracture of the catheter, catheter dislocation and embolization, pinch-off syndrome, and thrombosis of the internal jugular vein [1, 7].

Catheter dislodged from the infusion port migrating or embolizing to the heart is uncommon with an estimated frequency of 0.1%–1.8% but is a potentially dangerous complication [2–4, 8]. Catheter fracture and migration after internal jugular vein placement are very rare [4].

Various mechanisms hypothesized for migration are forced flushing, vigorous movements of upper limbs, neck flexion, congestive heart failure, and change in thoracic pressure with coughing and vomiting [9].

The most frequent location of fracture at the proximal port anastomosis between the injection port and the catheter is 93.2% of cases, and the middle part is 6.8% [8]. The dislodged or fractured catheter fragment may embolise to the right atrium, ventricle, pulmonary artery, or internal jugular vein. Clinical presentations vary with symptoms of catheter malfunction, palpitations, cough, shortness of breath, or septic syndrome, and may complicate with myocardial perforation, perforation of the vein, migration into mediastinal structures, internal jugular vein thrombosis, and pulmonary artery pseudoaneurysm [5, 10]. Early detection of the complication is hence important for management. Early removal of the catheter fragment is recommended. Percutaneous endovascular techniques can be used to retrieve the catheter fragment [4, 11, 12].  Table 1 summarises the case reports of chemoport migration and dislodgement.

In our patient, the dislodged catheter had migrated to the pulmonary artery and was entrapped in the main pulmonary artery and the left pulmonary artery. Uncustomarily, our patient was asymptomatic and was successfully managed with the percutaneous retrieval of the catheter before the development of complications.

## 4. Conclusion

Catheter dislodgement and migration are rare but potentially life-threatening complications that must be recognized and treated promptly. Percutaneous transvenous retrieval of intravascular fractured port catheters is a safe procedure and may be recommended for the management of patients with fractured port catheters in situ.

## Figures and Tables

**Table 1:** Case report summary of chemoport migration and dislodgement.

**Author**	**Case details**			
	**Age/sex**	**Diagnosis**	**Course**	**Outcome**
Lakshmi et al. [13]	55 year/F	Infiltrating ductal carcinoma of the left breast	Fracture of chemoport catheter at the junction of first rib and clavicle with embolization of distal fragment into the heart	Retrieval under fluoroscopic guidance by snare via a transfemoral approach
	42 year/F	Infiltrating duct carcinoma of the right breast	Fractured port catheter with distal segment located in superior segmental branch of right pulmonary artery	Retrieved using a snare via a transfemoral approach
	48 year/F	Infiltrating ductal carcinoma of the left breast	Fracture of chemoport with distal fragment embolized into the heart	Retrieved under fluoroscopic guidance by using a snare via a transfemoral approach

Goyal et al. [14]	3 year/F	B cell ALL	Chemoport migration to RV	Endovascular retrieval failed. Surgical removal of the migrated catheter

Chuah et al. [15]	50 year/M	Stage III descending colon carcinoma	Pinch-off syndrome with embolization of the distal half of the catheter to the right ventricle	Removal of the fractured catheter via percutaneous endovascular approach under fluoroscopic guidance

A. Agarwal and M. Agarwal [16]	56 year/F	Acute lymphoblastic leukemia	The long tubular intravascular part of the catheter of approximately 11 cm had migrated to the right ventricle and right atrium	The fragmented catheter was removed using a gooseneck snare technique

## Data Availability

The data that support the findings of this study are available from the corresponding author upon reasonable request.
